# Transformation from e-voting to e-cheque

**DOI:** 10.1371/journal.pone.0302659

**Published:** 2024-06-20

**Authors:** Yun-Xing Kho, Swee-Huay Heng, Syh-Yuan Tan, Ji-Jian Chin

**Affiliations:** 1 Faculty of Information Science and Technology, Multimedia University, Melaka, Malaysia; 2 School of Engineering, Computing and Mathematics, University of Plymouth, Plymouth, England, United Kingdom; IMT Alti Studi: Scuola IMT Alti Studi Lucca, ITALY

## Abstract

Although e-voting scheme and e-cheque scheme are two different applications, they have similarities in the scheme definitions and security properties. This inspires us to establish a relationship between the two schemes by formalising a generic transformation from e-voting to e-cheque scheme. Firstly, we define the scheme definitions and security models for both e-voting scheme and e-cheque scheme. Subsequently, we demonstrate a generic transformation framework from e-voting to e-cheque with asymptotic complexity of O(n) and design a formal proof to show that a secure e-voting scheme can be transformed into a secure e-cheque scheme. As a proof of concept, we apply our newly proposed transformation technique to the e-voting scheme proposed by Li et al. and obtain a concrete e-cheque scheme.

## 1 Introduction

There are many studies of electronic systems (e-systems) in the literature such as e-voting [1–4] and e-cheque [5–8]. Chaum in 1981 first introduced the concept of e-voting scheme [9] that serves as a platform that permits an individual to collaboratively make a decision or to choose a representative through online means while Chaum et al. in 1988 [10] first introduced the concept of e-cheque scheme in which e-cheque is a digital analogy to a paper cheque. Even though these systems may seem very different in their respective applications, they share similarities in their scheme definitions and security properties which lead to the possibility of establishing a generic transformation framework, that is we can derive one scheme from another scheme. However, the research communities are disjointed [11] and to the best of our knowledge, there is no transformation frameworks between e-voting and e-cheque have been explored. The beauty of transformation is that we do not need to build the entire scheme from scratch, and a transformed scheme inherits the security guarantee from the original scheme.

### 1.1 Related work

**Fig 1:**
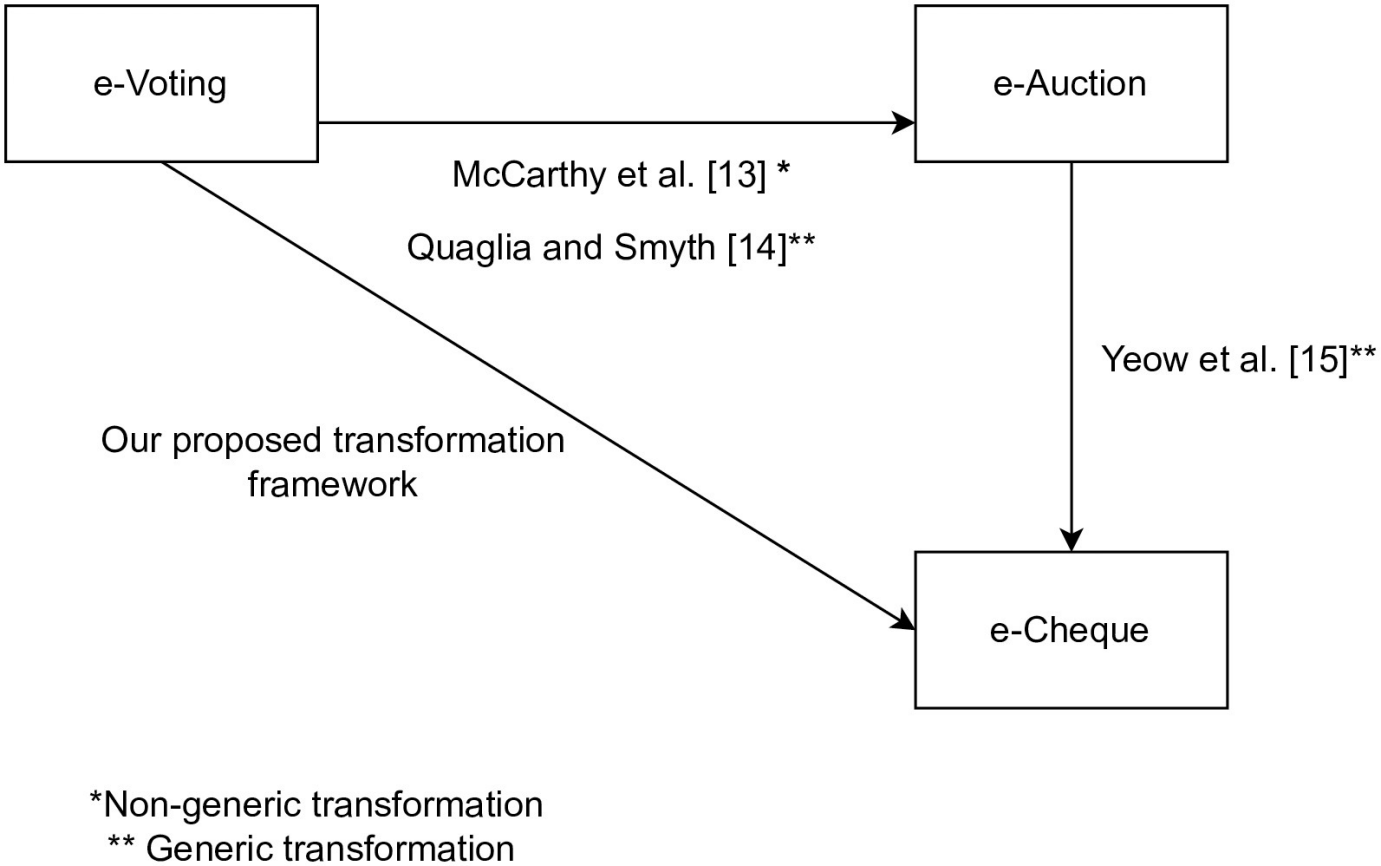
Transformation between e-auction, e-voting, and e-cheque.

e-Auction was first proposed by Franklin and Reiter in 1996 [12]. In an e-auction, the auctioneer can place products or services on the website for auction and the bidder can bid for their desired products or services on the bidding website. The bidder with the highest bid wins the game. McCarthy et al. [13] and Quaglia and Smyth [14] presented some transformations from e-voting to e-auction subsequently. More specifically, McCarthy et al. [13] proposed two specific transformations from e-voting to e-auction, namely, from Helios e-voting to Hawk e-auction scheme and from Civitas e-voting to Aucitas e-auction. McCarthy et al. [13] claimed that the Hawk e-auction satisfied indistinguishability under chosen-plaintext attack (IND-CPA) while the Aucitas e-auction satisfied collusion resistance without providing security proofs [14]. Quaglia and Smyth [14] proposed a generic transformation framework from e-voting to a secret, verifiable e-auction. Quaglia and Smyth [14] revised the proposed scheme of McCarthy et al. [13] by providing strong theoretical foundation where the scheme satisfied correctness, injectivity, completeness, verifiability and bid secrecy. Yeow et al. [15] presented a generic transformation framework from e-auction to e-cheque. Their proposed transformation framework satisfied existential unforgeability under chosen account attack (EUF-CAA), payer anonymity under chosen account attack (PA-CAA), and indistinguishability under chosen cheque attack (IND-CCeA). We observed that since e-voting can be transformed into e-auction and e-auction can be transformed into e-cheque as shown in Fig 1. To the best of our knowledge, there is no direct transformation from e-voting to e-cheque has been proposed in the literature. Hence, it would be natural to explore the possibility of direct transformation between e-voting and e-cheque as the two schemes possess high similarities in terms of scheme definitions and security properties. In this work, we demonstrate that the disjoint research fields of e-voting and e-cheque are related. Our work unifies e-voting and e-cheque, and thus expedite the development of both fields. Particularly, a secure e-cheque scheme can now be directly derived from an e-voting scheme without first transforming the e-voting to an e-auction and then only transforming the e-auction to an e-cheque.

While Quaglia and Smyth [14] proposed a generic transformation framework from e-voting to e-auction with asymptotic complexity of O(n), Yeow et al. [15] presented a generic transformation framework from e-auction to e-cheque with asymptotic complexity of O(n). Therefore, using current transformation frameworks to obtain e-cheque from e-voting scheme required first transforming the e-voting to an e-auction and then only transforming the e-auction to an e-cheque, thus required 2O(n) as shown in Table 1. We propose a direct transformation from e-voting to e-cheque which only required the complexity of O(n).

**Table 1:** Comparison with related generic transformation frameworks.

Scheme	Transformation Framework	Complexity
Quaglia and Smyth [14]	From e-voting to e-auction	O(n)
Yeow et al. [15]	From e-auction to e-cheque	O(n)
Our Work	From e-voting to e-cheque	O(n)

### 1.2 Our contribution

In this paper, we first review the scheme definitions of e-voting and e-cheque, followed by their security models respectively. While a rigorous security model for e-cheque schemes has been established [15], it is not the case for e-voting schemes. Therefore, we define some important security properties for e-voting, namely, confidentiality, anonymity, and unforgeability that are required to perform the transformation before presenting the generic transformation from e-voting to e-cheque. With that, we can support the proposed transformation with rigorous security proofs which shows that if the underlying e-voting scheme fulfills confidentiality, anonymity, and unforgeability, then the transformed e-cheque scheme is also fulfills confidentiality, anonymity, and unforgeability. Finally, we demonstrate this established transformation framework by providing an instance in which we exhibit how to derive an e-cheque scheme by employing the e-voting scheme proposed by Li et al. [16] as the underlying scheme.

## 2 Definitions

### 2.1 e-voting

Since the existing definitions for e-voting schemes are more specific based on the respective constructions, we make an effort to provide a more general definition which applies to all construction.

The e-voting scheme consists of three algorithms:

- **Register (1^*k*^) → {(*pk*_*T*_, *sk*_*T*_), (*pk*_*V*_, *sk*_*V*_)}**: This algorithm is executed by a trusted third party (TTP). It takes the security parameters 1^*k*^ as the input and outputs a pair of public and private keys for the tallier (*pk*_*T*_, *sk*_*T*_) and the voter (*pk*_*V*_, *sk*_*V*_).
- **Vote (*pk*_*T*_, *sk*_*V*_, *v*) → (*Bal*)**: This algorithm is executed by the voter. It takes the tallier’s public key *pk*_*T*_, the voter’s private key *sk*_*V*_, and the voter’s choice of candidates (*v*) as input and outputs ballot (*Bal*). The voter submits *Bal* to the tallier to cast a vote.
- **Tally (*sk*_*T*_, *pk*_*V*_, *Bal*) → (*Result*_*V*_)**: This algorithm is executed by the tallier. The tallier takes the tallier’s private key *sk*_*T*_, the voter’s public key *pk*_*V*_, and the ballot *Bal* as input, verifies if the *Bal* is valid then computes the tally result (*Result*_*V*_) of the valid *Bal*.

### 2.2 e-cheque

The e-cheque scheme consists of three algorithms [15]:

- **Register (1^*k*^) → {(*pk*_*B*_, *sk*_*B*_), (*pk*_*P*_, *sk*_*P*_)}**: This algorithm is executed by a trusted third party (TTP). It takes the security parameters 1^*k*^ as input and outputs a pair of public and private keys for the bank (*pk*_*B*_, *sk*_*B*_) and the payer (*pk*_*P*_, *sk*_*P*_).
- **Write (*pk*_*B*_, *sk*_*P*_, *M*) → (*ϑ*)**: This algorithm is executed by the payer. It takes the bank’s public key *pk*_*B*_, the payer’s private key *sk*_*P*_, and *M* where *M* = (*I*, $), *I* is the account information and $ is the amount as input and outputs a concealed cheque (*ϑ*). The payer submits *ϑ* to the bank system.
- **Transfer (*sk*_*B*_, *pk*_*P*_, *ϑ*) → (*Result*_*T*_)**: This algorithm is executed by the bank. The bank takes its own private key *sk*_*B*_, payer’s public key *pk*_*P*_, and a concealed cheque *ϑ* as input and verifies if the *ϑ* is valid then the bank processes the transaction (*Result*_*T*_) according to the *M* embedded in valid *ϑ*.

## 3 Security model

### 3.1 Security requirements for e-voting

1. **Confidentiality**. According to Bernhard et al. [17], confidentiality and privacy are synonymous in most security applications. In an e-voting scheme, privacy means the cast votes are anonymous to any party except when the election result reveals the vote [18]. We define for the first time the following game as indistinguishability under chosen ballot attack (IND-CBAA). We define the game between the Adversary and Challenger as follows.
  - Registration phase: The Challenger provides the system parameters to the Adversary.
  - Training phase: The Adversary can query *v*_*i*_ to the Vote oracle and get a ballot *Bal*_*i*_ in return where *i* is the number of iterations run by the Adversary. The Adversary can verify *Bal*_*i*_ by issuing *Bal*_*i*_ to the Tally oracle. The Tally oracle will reply tally result to the Adversary, the Adversary extracts the validity result either valid or invalid from the tally result.
  - Identifying phase: The Adversary chooses *v*_0_ and *v*_1_ and sends both to the Challenger. The Challenger chooses a random *b* ∈ {0, 1} and returns Bal* where Bal* is generated from *v*_*b*_. The Adversary makes a guess *b*′ = {0, 1} and wins the game if *b*′ = *b*.
  **Definition 1 (IND-CBAA)**. *An e-voting scheme is* (*ε*, *t*)-*indistinguishable under chosen ballot attack (IND-CBAA) if no probabilistic polynomial time Adversary* A *can win the game above in time t*, *Adversaries advantage ε*, *and* $\text{Pr}\left[b^{\prime }=b\right]\leq \frac{1}{2}+\varepsilon$.
2. **Anonymity**. According to Zaghloul et al. [1], anonymity in an e-voting scheme means the identity of the voter remains anonymous. We define for the first time the following game as indistinguishability under chosen voter’s vote attack (IND-CVA). We define the game between the Adversary and Challenger as follows.
  - Registration phase: The Challenger provides the system parameters to the Adversary.
  - Training phase: The Adversary can query *v*_*i*_ to the Vote oracle and get a ballot *Bal*_*i*_ in return where *i* is the number of iterations run by the Adversary. The Adversary can verify *Bal*_*i*_ by issuing *Bal*_*i*_ to the Tally oracle. The Tally oracle will reply tally result to the Adversary, the Adversary extracts the validity result either valid or invalid from the tally result.
  - Identifying phase: The Adversary chooses v* and sends it to the Challenger. The Challenger returns *Bal*_*b*_ where *b* ∈ {0, 1} and one of them is generated by using v*. The Adversary makes a guess *b*′ = {0, 1} and wins the game if *b*′ = *b*.
  **Definition 2 (IND-CVA)**. *An e-voting scheme is* (*ε*, *t*)-*indistinguishable under chosen voter’s vote attack (IND-CVA) if no probabilistic polynomial time Adversary* A *can win the game above in time t*, *Adversaries advantage ε*, *and* $\text{Pr}\left[b^{\prime }=b\right]\leq \frac{1}{2}+\varepsilon$.
3. **Unforgeability**. According to Li and Lai [19], unforgeability in an e-voting scheme means it is infeasible to forge a valid ballot for another voter. We define for the first time the following game as existential unforgeability under chosen vote attack (EUF-CVA). We define the game between the Adversary and Challenger as follows.
  - Registration phase: The Challenger provides the system parameters to the Adversary.
  - Training phase: The Adversary can query *v*_*i*_ to the Vote oracle and get a ballot *Bal*_*i*_ in return where *i* is the number of iterations run by the Adversary. The Adversary can verify *Bal*_*i*_ by issuing *Bal*_*i*_ to the Tally oracle. The Tally oracle will reply tally result to the Adversary, the Adversary extracts the validity result either valid or invalid from the tally result.
  - Forging phase: The Adversary chooses v* and forges Bal*. If the Bal* is a valid ballot, the Adversary wins the game.
  **Definition 3 (EUF-CVA)**. *An e-voting scheme is* (*ε*, *t*)-*existential unforgeable under chosen vote attack (EUF-CVA) if no probabilistic polynomial time Adversary* A *can win the game above in time t*, *Adversaries advantage ε*, *and* Pr[Bal* *is* *valid*] ≤ *ε*.

### 3.2 Security requirements of e-cheque

1. **Confidentiality**. According to Yeow et al. [15], confidentiality in the e-cheque scheme means the invalid and unused e-cheques are anonymous to any party. The following game is the indistinguishability under chosen cheque attack (IND-CCEA) security notion for an e-cheque scheme. The security model proposed by Yeow et al. [15] is as follows.
  - Registration phase: The Challenger provides the system parameters to the Adversary.
  - Training phase: The Adversary can query *M*_*i*_ to the Write oracle and get a cheque *ϑ*_*i*_ in return where *i* is the number of iterations the Adversary runs. The Adversary can verify *ϑ*_*i*_ by issuing *ϑ*_*i*_ to the Transfer oracle. The Transfer oracle will reply transaction result to the Adversary, the Adversary extracts the validity result either valid or invalid from the transaction result.
  - Identifying phase: The Adversary chooses *M*_0_ and *M*_1_ and sends both to the Challenger. The Challenger chooses a random *b* ∈ {0, 1} and returns ϑ* where ϑ* is generated from *M*_*b*_. The Adversary makes a guess $b^{\prime }=\left\{0,1\right\}$ and wins the game if $b^{\prime }=b$.
  **Definition 4 (IND-CCEA)**. *An e-cheque scheme is* (*ε*′, *t*)-*indistinguishable under chosen cheque attack (IND-CCEA) if no probabilistic polynomial time Adversary* A *can win the game above in time t*, *Adversaries advantage ε, and* $\text{Pr}\left[b^{\prime }=b\right]\leq \frac{1}{2}+\varepsilon^{\prime }$.
2. **Anonymity**. According to Yeow et al. [15], anonymity in the e-cheque scheme means the identity of the payer remains secret from others except for the bank. The following game is the indistinguishability under chosen cheque’s information attack (IND-CIA) security notion for an e-cheque scheme. The security model proposed by Yeow et al. [15] is as follows.
  - Registration phase: The Challenger provides the system parameters to the Adversary.
  - Training phase: The Adversary can query *M*_*i*_ to the Write oracle and get a cheque *ϑ*_*i*_ in return where *i* is the number of iterations the Adversary runs. The Adversary can verify *ϑ*_*i*_ by issuing *ϑ*_*i*_ to the Transfer oracle. The Transfer oracle will reply transaction result to the Adversary, the Adversary extracts the validity result either valid or invalid from the transaction result.
  - Identifying phase: The Adversary chooses M* and sends it to the Challenger. The Challenger returns *ϑ*_*b*_ where *b* ∈ {0, 1} and one of them is generated by using M*. The Adversary makes a guess $b^{\prime }=\left\{0,1\right\}$ and wins the game if $b^{\prime }=b$.
  **Definition 5 (IND-CIA)**. *An e-cheque scheme is* (*ε*′, *t*)-*indistinguishable under chosen cheque’s information attack (IND-CIA) if no probabilistic polynomial time Adversary* A *can win the game above in time t*, *Adversaries advantage ε*, *and* $\text{Pr}\left[b^{\prime }=b\right]\leq \frac{1}{2}+\varepsilon^{\prime }$.
3. **Unforgeability**. According to Yeow et al. [15], unforgeability in an e-cheque scheme means it is infeasible to forge a valid signed e-cheque of another user. The following game is the existential unforgeability under chosen cheque’s information attack (EUF-CIA) security notion for an e-cheque scheme. The security model proposed by Yeow et al. [15] is as follows.
  - Registration phase: The Challenger provides the system parameters to the Adversary.
  - Training phase: The Adversary can query *M*_*i*_ to the Write oracle and get a cheque *ϑ*_*i*_ in return where *i* is the number of iterations the Adversary runs. The Adversary can verify *ϑ*_*i*_ by issuing *ϑ*_*i*_ to the Transfer oracle. The Transfer oracle will reply transaction result to the Adversary, the Adversary extracts the validity result either valid or invalid from the transaction result.
  - Forging phase: The Adversary chooses M* and forges ϑ*. If the ϑ* is a valid cheque, the Adversary wins the game.
  **Definition 6 (EUF-CIA)**. *An e-cheque scheme is* (*ε*′, *t*)-*existential unforgeable under chosen cheque’s information attack (EUF-CIA) if no probabilistic polynomial time Adversary* A *can win the game above in time t*, *Adversaries advantage ε, and* $\text{Pr}\left[\vartheta^{*}\;is\;valid\right]\leq \varepsilon^{\prime }$.

## 4 Transformation

We now present a generic transformation from e-voting scheme to an e-cheque scheme. We first explain the entities and associated information at below:

- Tallier in e-voting scheme plays the role as the bank in e-cheque scheme.
- Voter in e-voting scheme plays the role as the payer in e-cheque scheme.
- Candidate in e-voting scheme plays the role as the payee in e-cheque scheme.
- Ballot in e-voting scheme is viewed as the cheque in e-cheque scheme.
- Vote in e-voting scheme is viewed as the account information and amount in e-cheque scheme.

Subsequently, a transformed e-cheque scheme can be constructed as follows:

- **Register (1^*k*^) → {(*pk*_*B*_, *sk*_*B*_), (*pk*_*P*_, *sk*_*P*_)}**. A TTP runs the registration of e-voting Register (1_*k*_) → {(*pk*_*T*_, *sk*_*T*_), (*pk*_*V*_, *sk*_*V*_)} to set the (*pk*_*B*_, *sk*_*B*_) = (*pk*_*T*_, *sk*_*T*_) and (*pk*_*P*_, *sk*_*P*_) = (*pk*_*V*_, *sk*_*V*_).
- **Write (*pk*_*B*_, *sk*_*P*_, *M*) → *ϑ***. A payee runs the voting algorithm of e-voting Vote (*pk*_*T*_, *sk*_*V*_, *v*) → *Bal*, where *pk*_*T*_ = *pk*_*B*_, *sk*_*V*_ = *sk*_*P*_, *v* = *M*, and the output *Bal* = *ϑ*.
- **Transfer (*sk*_*B*_, *pk*_*P*_, *ϑ*) → *Result*_*T*_**. The bank runs the tally algorithm of the e-voting Tally (*sk*_*T*_, *pk*_*V*_, *Bal*) → *Result*_*V*_, where *sk*_*T*_ = *sk*_*B*_, *pk*_*V*_ = *pk*_*P*_, *Bal* = *ϑ*, and the verification result of the e-voting *Result*_*V*_ = the verification result of the e-cheque *Result*_*T*_

We note that there exists an implementation process to which we may need to pay more attention. More specifically, a bulletin board is required in an e-voting scheme but it is not required in an e-cheque scheme. Therefore, we propose to treat the bulletin board in the e-voting scheme as a platform in the banking system to verify the status of the cheque transaction process, which somehow seems natural.

We also noticed that the transformation from e-cheque to e-voting cannot be performed directly due to the security requirements for e-voting are more stringent than e-cheque. In precise, an e-voting scheme requires receipt-freeness, where the voter cannot attain any information that can be used to prove how he voted for any party. It also demands coercion-resistance, where the coercers cannot insist that voters vote in a certain way and the voter cannot prove his vote to the information buyer [20]. On the contrary, e-cheque scheme does not require these properties. Nevertheless, extensive studies are required to affirm if such a transformation is possible.

## 5 Security analysis

We provide the security analysis to show that the transformed e-cheque scheme fulfils the respective security requirements which follow directly from those of the underlying e-voting scheme. Theorem 1, Theorem 2, and Theorem 3 present respectively the security analysis of confidentiality, anonymity and unforgeability of the transformed e-cheque scheme from e-voting scheme.

### 5.1 Confidentiality

**Theorem 1**. *Let e-voting* = {*Register, Vote, Tally*} *be the secure e-voting scheme and let e-cheque* = {*Register, Write, Transfer*} *be the transformed e-cheque scheme. If the underlying e-voting scheme is* (*t*, *q*_*v*_, *ε*)-*secure against indistinguishability under chosen ballot attack (IND-CBAA), then the transformed e-cheque scheme is* (*t*′, *q*_*w*_, *ε*′)-*secure against indistinguishability under chosen cheque attack (IND-CCEA), where*

$$\begin{array}{c} t=t^{\prime },q_{v}=q_{w},\varepsilon =\varepsilon^{\prime }\leq \frac{1}{2}+n\left(k\right) \end{array}$$ (1)

*q*_*w*_, *q*_*v*_ *are the total write and vote query, respectively, and n is a negligible function parameterised by the security parameter k*.

**Fig 2:**
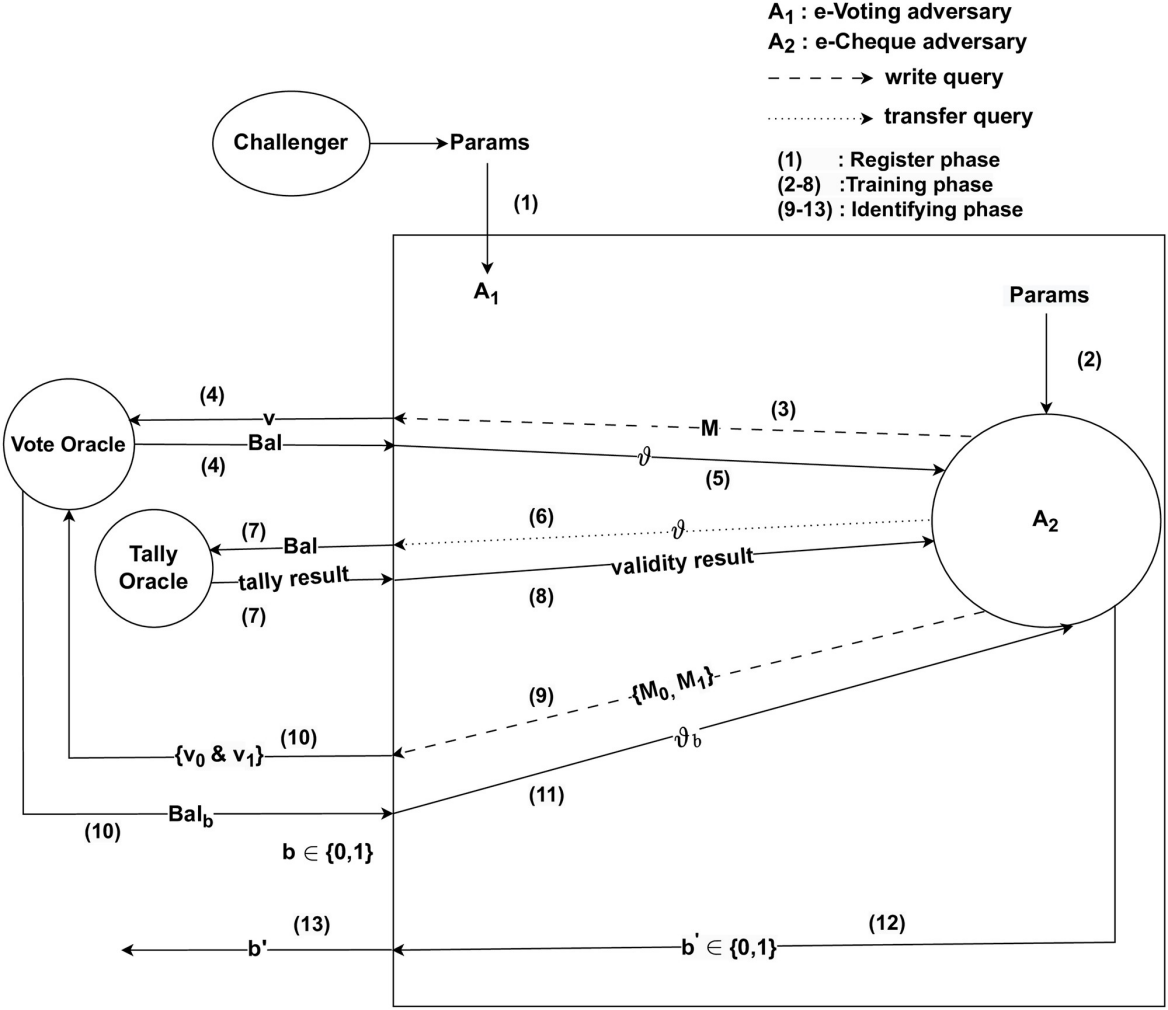
Proof of contradiction—Confidentiality.

*Proof.* Suppose that *A*_2_ is an Adversary who (*t*′, *q*_*w*_, *ε*′)-breaks the IND-CCEA of e-cheque scheme. We show that e-voting scheme is not (*t*, *q*_*v*_, *ε*)- secure. Hence, we show how *A*_1_ can use *A*_2_ to (*t*, *q*_*v*_, *ε*)-break the IND-CBAA of e-voting scheme. *A*_1_ runs *A*_2_ as a subroutine and simulates its attack environment. Fig 2 shows the simulated Adversary game and the environment between *A*_1_ and *A*_2_.

The Challenger passes *Params* to *A*_1_. *A*_1_ passes *Params* to *A*_2_ and completed the Register phase. In the Training phase, *A*_2_ issues *M* as a write query to *A*_1_. *A*_1_ sets *v* = *M* and inputs *v* to Vote oracle using vote query to produce *Bal*. *A*_1_ sets *ϑ* = *Bal*, *A*_1_ returns *ϑ* to *A*_2_. *A*_2_ issues *ϑ* as a transfer query to *A*_1_. *A*_1_ sets *Bal* = *ϑ* and inputs *Bal* to Tally oracle to verify if *Bal* is valid. The Tally oracle returns the tally result to *A*_1_, *A*_1_ extracts the validity result from the tally result and returns the validity result either valid or invalid to *A*_2_.

At some point, *A*_2_ decides that the Training phase is over and starts the Identifying phase. *A*_2_ chooses *M*_0_ and *M*_1_. *A*_2_ passes *M*_0_ and *M*_1_ to *A*_1_. *A*_1_ sets *v*_0_ = *M*_0_, *v*_1_ = *M*_1_. *A*_1_ sends *v*_0_ and *v*_1_ to Vote oracle to obtain *Bal*_*b*_. *A*_1_ sets *ϑ*_*b*_ = *Bal*_*b*_, *A*_1_ delivers *ϑ*_*b*_ as the problem in IND-CBAA as the challenge to *A*_2_. With a probability $\varepsilon^{\prime }\leq \frac{1}{2}+n\left(k\right)$, *A*_2_ outputs a correct guess *b*′ in return. *A*_1_ uses *A*_2_’s answer as its guess. Since *b*′ = *b*, *A*_1_ thus breaks IND-CBAA security.

As *A*_1_ simulates the environment perfectly, we have *ε* = *ε*′ and *t* = *t*′ as required where *A*_1_ runs in time *t* while *A*_2_ runs in time *t*′.

### 5.2 Anonymity

**Theorem 2**. *Let e-voting* = {*Register, Vote, Tally*} *be the secure e-voting scheme and let e-cheque* = {*Register, Write, Transfer*} *be the transformed e-cheque scheme. If the underlying e-voting scheme is* (*t*, *q*_*v*_, *ε*)-*secure against indistinguishability under chosen voter’s vote attack (IND-CVA), then the transformed e-cheque scheme is* (*t*′, *q*_*w*_, *ε*′)-*secure against indistinguishability under chosen cheque’s information attack (IND-CIA), where*

$$\begin{array}{c} t=t^{\prime },q_{v}=q_{w},\varepsilon =\varepsilon^{\prime }\leq \frac{1}{2}+n\left(k\right) \end{array}$$ (2)

*q*_*w*_, *q*_*v*_ *is the total write and vote query, respectively, and n is a negligible function parameterised by the security parameter k*.

**Fig 3:**
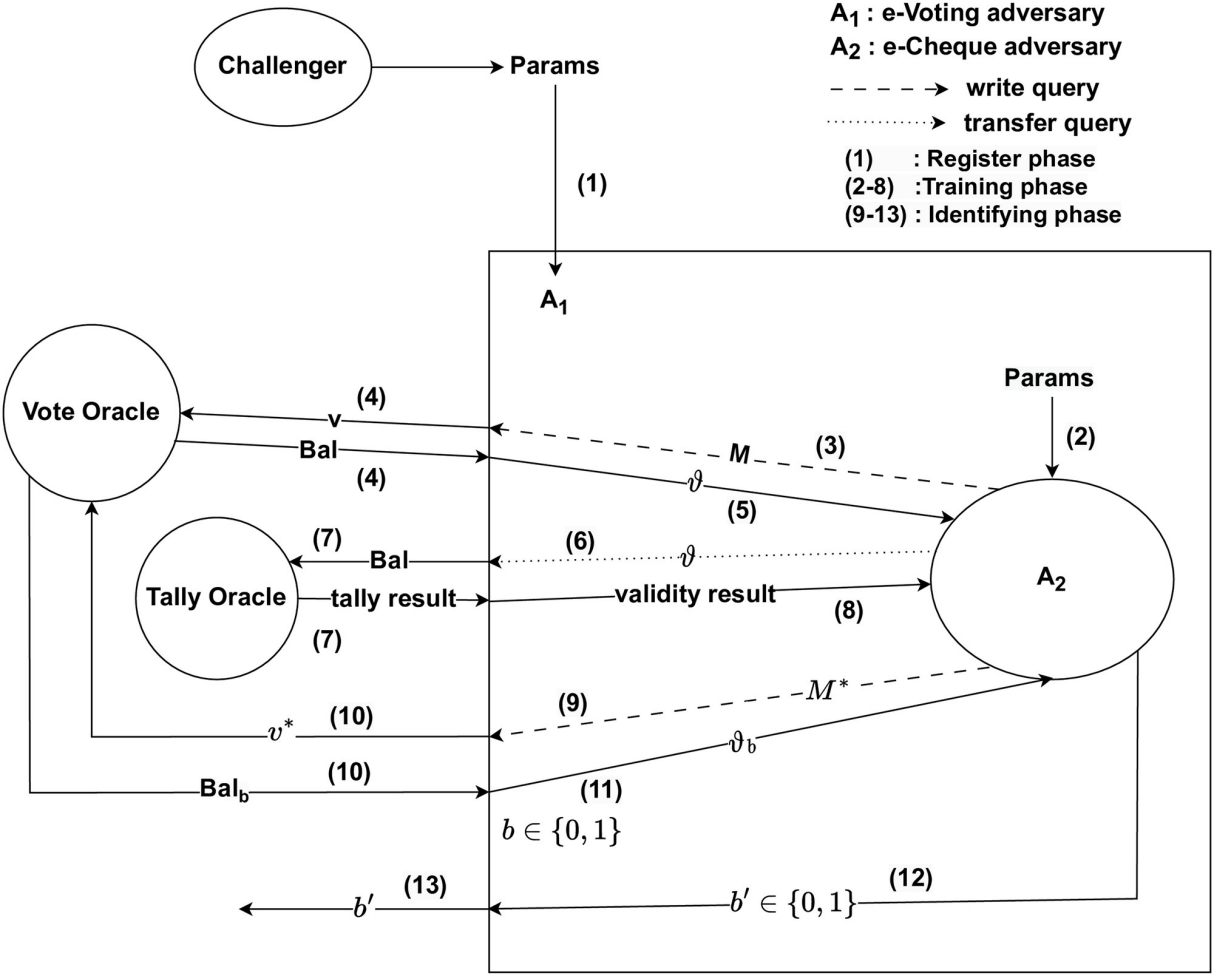
Proof of contradiction—Anonymity.

*Proof.* Suppose that *A*_2_ is an Adversary who (*t*′, *q*_*w*_, *ε*′)-breaks the IND-CIA of e-cheque scheme. We show that e-voting scheme is not (*t*, *q*_*v*_, *ε*)- secure. Hence, we show how *A*_1_ can use *A*_2_ to (*t*, *q*_*v*_, *ε*)-break the IND-CVA of e-voting scheme. *A*_1_ runs *A*_2_ as a subroutine and simulates its attack environment. Fig 3 shows the simulated adversarial game and environment between *A*_1_ and *A*_2_.

The Challenger passes *Params* to *A*_1_. *A*_1_ passes *Params* to *A*_2_ and completed the Register phase. In the Training phase, *A*_2_ issues *M* as a write query to *A*_1_. *A*_1_ sets *v* = *M* and inputs *v* to Vote oracle using vote query to produce *Bal*. *A*_1_ sets *ϑ* = *Bal*, *A*_1_ returns *ϑ* to *A*_2_. *A*_2_ issues *ϑ* as a transfer query to *A*_1_. *A*_1_ sets *Bal* = *ϑ* and inputs *Bal* to Tally oracle to verify if *Bal* is valid. The Tally oracle returns the tally result to *A*_1_, *A*_1_ extracts the validity result from the tally result and returns the validity result either valid or invalid to *A*_2_.

At some point, *A*_2_ decides that the Training phase is over and starts the Identifying phase. *A*_2_ passes M* to *A*_1_. *A*_1_ sets v* = M* and sends v* to Vote oracle to obtain *Bal*_*b*_ where *b* ∈ {0, 1} and one of them is generated by using v*. *A*_1_ sets *ϑ*_*b*_ = *Bal*_*b*_ and returns *ϑ*_*b*_ as the problem in IND-CVA as the challenge to *A*_2_. With a probability $\varepsilon^{\prime }\leq \frac{1}{2}+n\left(k\right)$, *A*_2_ outputs a correct guess *ϑ*_*b*_ in return. *A*_1_ uses *A*_2_’s answer as its guess. Since *ϑ*_*b*_ is valid, then *Bal*_*b*_ is valid, *A*_1_ thus breaks IND-CVA security.

As *A*_1_ simulates the environment perfectly, we have *ε* = *ε*′ and *t* = *t*′ as required where *A*_1_ runs in time *t* while *A*_2_ runs in time *t*′.

### 5.3 Unforgeability

**Theorem 3**. *Let e-voting* = {*Register, Vote, Tally*} *be the secure e-voting scheme and let e-cheque* = {*Register, Write, Transfer*} *be the transformed e-cheque scheme. If the underlying e-voting scheme is* (*t*, *q*_*v*_, *ε*)-*secure against existential unforgeable under chosen vote attack (EUF-CVA), then the transformed e-cheque scheme is* (*t*′, *q*_*w*_, *ε*′)-*secure against existential unforgeability under chosen cheque’s information attack (EUF-CIA), where*

$$\begin{array}{c} t=t^{\prime },q_{v}=q_{w},\varepsilon =\varepsilon^{\prime }\leq n\left(k\right) \end{array}$$ (3)

*q*_*w*_, *q*_*v*_ *is the total write and vote query, respectively, and n is a negligible function parameterised by the security parameter k*.

**Fig 4:**
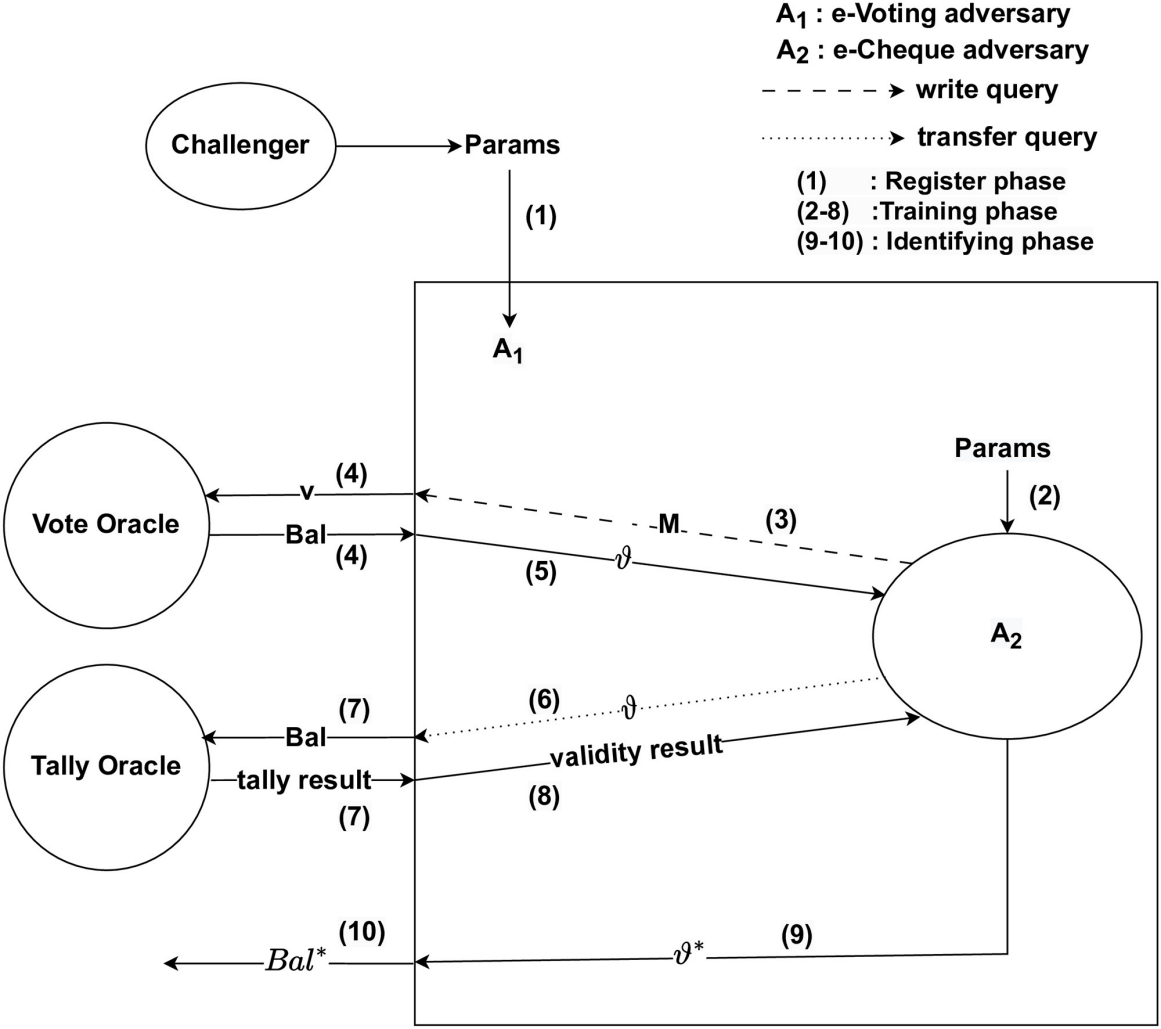
Proof of contradiction—Unforgeability.

*Proof.* Suppose that *A*_2_ is an Adversary who (*t*′, *q*_*w*_, *ε*′)-breaks the EUF-CIA of e-cheque scheme. We show that e-voting scheme is not (*t*, *q*_*v*_, *ε*)- secure. Hence, we show how *A*_1_ can use *A*_2_ to (*t*, *q*_*v*_, *ε*)-break the EUF-CVA of e-voting scheme. *A*_1_ runs *A*_2_ as a subroutine and simulates its attack environment. Fig 4 shows the simulated adversarial game and environment between *A*_1_ and *A*_2_.

The Challenger passes *Params* to *A*_1_. *A*_1_ passes *Params* to *A*_2_ and completed the Register phase. In the Training phase, *A*_2_ issues *M* as a write query to *A*_1_. *A*_1_ sets *v* = *M* and inputs *v* to Vote oracle using vote query to produce *Bal*. *A*_1_ sets *ϑ* = *Bal*, *A*_1_ returns *ϑ* to *A*_2_. *A*_2_ issues *ϑ* as a transfer query to *A*_1_. *A*_1_ sets *Bal* = *ϑ* and inputs *Bal* to Tally oracle to verify if *Bal* is valid. The Tally oracle returns the tally result to *A*_1_, *A*_1_ extracts the validity result from the tally result and returns the validity result either valid or invalid to *A*_2_.

At some point, *A*_2_ decides that the Training phase is over and starts the Forging phase. With a probability $\varepsilon^{\prime }\leq n\left(k\right)$, *A*_2_ outputs a guess ϑ* to *A*_1_. *A*_1_ uses *A*_2_’s answer as its guess. Since ϑ* is valid, then Bal* is valid, *A*_1_ thus breaks EUF-CVA security.

As *A*_1_ simulates the environment perfectly, we have *ε* = *ε*′ and *t* = *t*′ as required where *A*_1_ runs in time *t* while *A*_2_ runs in time *t*′.

## 6 An instance

Li et al. [16] proposed an anonymous authentication scheme, namely, event-oriented linkable and traceable anonymous authentication (EOLTAA) and utilised the EOLTAA scheme with public key encryption scheme that is semantically secure to construct a blockchain e-voting scheme. We provide a review of the underlying PKE, EOLTAA and the scheme definitions of Li et al.’s e-voting scheme. We then formally prove that their proposed e-voting scheme possesses confidentiality, anonymity, and unforgeability. Lastly, we perform a transformation from Li et al.’s e-voting scheme to an e-cheque scheme as an instance.

### 6.1 Underlying cryptographic tools

We review the public key encryption scheme and event-oriented linkable and traceable anonymous authentication scheme as follows.

- Public Key Encryption (PKE) Scheme [21]. A PKE scheme consists of three algorithms:
  - E.KeyGen (1^λ^) → (*pk*_*e*_, *sk*_*e*_). This algorithm takes security parameter (1^λ^) as the input and outputs a pair of public and private key for the user (*pk*_*e*_, *sk*_*e*_).
  - E.Encrypt (*m*, *pk*_*e*_) → *C*. This algorithm takes a message *m* and a public key (*pk*_*e*_) as input and outputs a ciphertext *C*.
  - E.Decrypt (*C*, *sk*_*e*_) → *m*. This algorithm takes a ciphertext *C* and a private key *sk*_*e*_ as input and outputs the message *m*.
- The Event-Oriented Linkable and Traceable Anonymous Authentication (EOLTAA) Scheme [16]. An EOLTAA scheme contains seven algorithms:
  - CSetup (1^λ^) → (*MSK*, *MPK*). The master key generation algorithm takes security parameter (λ) as input and outputs a master secret key (*MSK*) and a master public key (*MPK*).
  - UKeyGen (1^λ^) → (*usk*, *upk*). The user key generation algorithm takes a security parameter (λ) as input and outputs a secret key (*usk*) and a public key (*upk*).
  - CertGen (*upk*, *MSK*) → *Cert*. The certificate generation algorithm takes *upk* and *MSK* as input and outputs a certificate (*Cert*) that validates the corresponding *upk*.
  - Auth (*m* = *e* ∥ *p*, *upk*, *usk*, *Cert*, *MPK*) → *π*. The authentication algorithm takes message (*m*), event identifier (*e*), payload (*p*), *upk*, *usk*, *Cert*, *MPK* as input and outputs an authentication token (*π*) on the *m*.
  - Verify (*m*, *π*, *MPK*) → 0/1. The verification algorithm takes *m*, *π*, *MPK* as input and outputs 0 or 1 to verify if the proof is invalid or valid.
  - Link (*m*_1_, *m*_2_, *π*_1_, *π*_2_) → 0/1. The linking algorithm takes two valid *m*, and authentication token pairs (*m*_1_, *π*_1_), (*m*_2_, *π*_2_) as input and outputs 1 if the two *m* bind with a common event that is authenticated by the same user; otherwise, outputs 0.
  - Trace (*m*_1_, *m*_2_, *π*_1_, *π*_2_) → ⊥ /*upk*. The trace algorithm takes two valid *m*, and authentication token pairs (*m*_1_, *π*_1_), (*m*_2_, *π*_2_) as input and outputs *upk* of the user who authenticates two messages that bind with a common event; otherwise, outputs ⊥.

### 6.2 Li et al.’s e-voting scheme

We review the e-voting scheme definitions as follows.

Suppose *E* = {*E*.*KeyGen*, *E*.*Encrypt*, *E*.*Decrypt*} is a semantically secure public key encryption scheme and Φ = {Φ_⋅_*CSetup*, Φ_⋅_*UKeyGen*, Φ_⋅_*CertGen*, Φ_⋅_*Auth*, Φ_⋅_*Verify*, Φ_⋅_*Link*, Φ_⋅_*Trace*} is the event-oriented linkable and traceable anonymous authentication scheme. The e-voting scheme consists of four phases, namely, Setup, Register, Vote, and Tally.

**Setup 1^λ^ → *MPK*.** Certificate authority uses Φ.*CSetup* to generate master public key *MPK* and sends the public parameters as a transaction to the blockchain.

**Register 1^λ^ → (*pk*, *sk*);(*pk*, *MSK*) → *Cert*.** Voters and the tallier set up their key pairs using Φ_⋅_*UKeyGen* and register a certificate, *Cert* with certificate authority where the tallier holds {(*pk*_*T*_, *sk*_*T*_), *Cert*_*T*_} and the voters holds {(*upk*_*i*_, *usk*_*i*_), *Cert*_*i*_}.

**Vote (*pk*_*e*_, *v*) → *C*_*i*_; (*C*_*i*_, *upk*_*i*_, *usk*_*i*_, *Cert*_*i*_, *MPK*) → *π*_*i*_.** The tallier chooses a random number *vid* as e-voting’s ID and creates a key pair (*sk*_*e*_, *pk*_*e*_) used to encrypt the ballot. The tallier generates a new blockchain account address, *addr*_*T*_ and creates an authentication token, *π*_*T*_ to authenticate *vid*||*addr*_*T*_ where *π*_*T*_ = Φ_⋅_*Auth*(*vid*||*addr*_*T*_, *pk*_*T*_, *sk*_*T*_, *Cert*_*T*_, *MPK*). Tallier then creates a smart contract (*sc*) that consists of *vid*, *π*_*T*_, *pk*_*e*_, *MPK*. Tallier sends (*vid*||*addr*_*T*_, *sc*, *π*_*T*_) to blockchain using *addr*_*T*_.

After the voter receives this voting, the voter chooses one candidate and encrypts it with *pk*_*e*_ to generate *C*_*i*_. Voter creates an authentication token *π*_*i*_ = Φ_⋅_*Auth*(*vid*||*C*_*i*_, *upk*_*i*_, *usk*_*i*_, *Cert*_*i*_, *MPK*) and creates blockchain account address *addr*_*i*_. Voter sends (*C*_*i*_, *π*_*i*_) to the blockchain using *addr*_*i*_.

**Tally (*sk*_*e*_, *C*_*i*_, *π*_*i*_) → *result*.** Smart contract runs Φ_⋅_*Verify*(*vid*||*C*_*i*_, *π*_*i*_, *MPK*) to check each received ballot and its authentication pair. The invalid ballot is removed. Then, check if the valid ballot is double-vote by running Φ_⋅_*Link*(*C*_*i*_, C_*_, *π*_*i*_, π_*_) for each (C_*_, π_*_) that is used before, and run Φ_⋅_*Trace*(*C*_*i*_, C_*_, *π*_*i*_, π_*_) to detect the double-vote voter’s identity.

The tallier receives all valid ballots and decrypts them using (*sk*_*e*_) and calculates the final election result (*result*). The tallier generates zero-knowledge proof *π*_*result*_ with *sk*_*e*_ as the witness. In the end, the tallier sends {*result*, *π*_*result*_} to the blockchain and anyone can see the final election result.

### 6.3 The transformed e-cheque scheme

We perform a transformation from Li et al.’s [16] e-voting scheme to an e-cheque scheme as an instance. Li et al.’s e-voting scheme consists of four algorithms, namely, Setup, Register, Vote, Tally. We combine their Setup algorithm and Register algorithm to a single Register algorithm. We perform the transformation as follows.

Suppose *E* = {*E*.*KeyGen*, *E*.*Encrypt*, *E*.*Decrypt*} is a semantically secure public key encryption scheme and Φ = {Φ_⋅_*CSetup*, Φ_⋅_*UKeyGen*, Φ_⋅_*CertGen*, Φ_⋅_*Auth*, Φ_⋅_*Verify*, Φ_⋅_*Link*, Φ_⋅_*Trace*} is the event-oriented linkable and traceable anonymous authentication scheme. The e-cheque scheme consists of three phases, namely, Register, Write, and Transfer.

**Register 1^λ^ → (*MPK*, *pk*, *sk*);(*pk*, *MSK*) → *Cert*.** Certificate authority uses Φ.*CSetup* to generate master public key *MPK* and sends the public parameters as a transaction to the blockchain. Payer and the bank set up their key pairs using Φ_⋅_*UKeyGen* and register a certificate, *Cert* with certificate authority where the bank (*pk*_*B*_, *sk*_*B*_), *Cert*_*B*_ and payer (*upk*_*i*_, *usk*_*i*_), *Cert*_*i*_.

**Write (*pk*_*e*_, *M*) → *C*_*i*_; (*C*_*i*_, *upk*_*i*_, *usk*_*i*_, *Cert*_*i*_, *MPK*) → *π*_*i*_.** The bank chooses a random number *vid* as e-cheque’s ID and creates a key pair (*sk*_*e*_, *pk*_*e*_) used to encrypt the e-cheque. The bank generates a new blockchain account address, *addr*_*B*_ and creates an authentication token, *π*_*B*_ to authenticate *vid*||*addr*_*B*_ where *π*_*B*_ = Φ_⋅_*Auth*(*vid*||*addr*_*B*_, *pk*_*B*_, *sk*_*B*_, *Cert*_*B*_, *MPK*). bank then creates a smart contact (*sc*) that consists of *vid*, *π*_*B*_, *pk*_*e*_, *MPK*. bank sends (*vid*||*addr*_*B*_, *sc*, *π*_*B*_) to blockchain using *addr*_*B*_.

After the payer receives the e-cheque, the payer writes payee’s account information and amount *M* to the e-cheque and encrypts it with *pk*_*e*_ to generate *C*_*i*_. Payer creates an authentication token *π*_*i*_ = Φ_⋅_*Auth*(*vid*||*C*_*i*_, *upk*_*i*_, *usk*_*i*_, *Cert*_*i*_, *MPK*) and creates blockchain account address *addr*_*i*_. Payer sends (*C*_*i*_, *π*_*i*_) to the blockchain using *addr*_*i*_.

**Transfer (*sk*_*e*_, *C*_*i*_, *π*_*i*_) → *result*.** Smart contract runs Φ_⋅_*Verify*(*vid*||*C*_*i*_, *π*_*i*_, *MPK*) to check each received e-cheque and its authentication pair. The invalid e-cheque is removed. Then, check if the valid e-cheque is double-spent before by running Φ_⋅_*Link*(*C*_*i*_, C_*_, *π*_*i*_, π_*_) for each (C_*_, π_*_) that is used before, and runs Φ_⋅_*Trace*(*C*_*i*_, C_*_, *π*_*i*_, π_*_) to detect the double-spent payer’s identity.

The bank receives all valid cheque and decrypts them using (*sk*_*e*_) and credits the amount from payer’s account to payee’s account. The bank generates zero-knowledge proof *π*_*result*_ with *sk*_*e*_ as the witness. In the end, the bank notifies payer and payee that the e-cheque transaction is completed.

### 6.4 Security analysis of Li et al.’s e-voting scheme

Li et al. claimed that their e-voting scheme is secure since the underlying tools are secure without formal security analysis of the e-voting scheme. Thus, we formally prove that their proposed e-voting scheme possesses confidentiality, anonymity, and unforgeability, following our formalised security notions, namely, IND-CBAA, IND-CVA, and EUF-CVA.

#### 6.4.1 Confidentiality

**Theorem 4**. *Let AUTHPKE* = {*Register, Authentication, Verification*} *be the secure event-oriented linkable and traceable anonymous authentication scheme and public key encryption scheme. Let e-voting* = {*Register, Vote, Tally*} *be the e-voting scheme. If the underlying AUTHPKE scheme is* (*t*, *q*_*a*_, *ε*)-*secure against indistinguishability under chosen-ciphertext attacks (IND-CCA), then the e-voting scheme is* (*t*′, *q*_*v*_, *ε*′)-*secure against indistinguishability under chosen ballot attack (IND-CBAA), where*

$$\begin{array}{c} t=t^{\prime },q_{a}=q_{v},\varepsilon =\varepsilon^{\prime }\leq \frac{1}{2}+n\left(k\right) \end{array}$$ (4)

*q*_*v*_ *is the vote query*, *q*_*a*_ *is the authentication query, ε is the non-negligible advantage to break the IND-CCA in AUTHPKE*, *ε*′ *is the non-negligible advantage to break the IND-CBAA in e-voting*, *n is a negligible function parameterised by the security parameter k*, *and t is the time required to complete the attack*.

*Proof.* Suppose that *A*_2_ is an Adversary who (*t*′, *q*_*v*_, *ε*′)-breaks the IND-CBAA of e-voting scheme and *A*_1_ = *A*_*PKE*_ is the Adversary which (*t*, *q*_*a*_, *ε*)-breaks the IND-CCA of the AUTHPKE scheme. We show that AUTHPKE scheme is not (*t*, *q*_*a*_, *ε*)- secure. Hence, we show how *A*_1_ can use *A*_2_ to (*t*, *q*_*a*_, *ε*)-break the IND-CCA of AUTHPKE scheme. *A*_1_ runs *A*_2_ as a subroutine and simulates its attack environment.

The AUTHPKE Challenger passes *Params*, public key *upk*, private key *usk*, and certificate *Cert* to *A*_1_ where the *upk*, *usk*, and *Cert* are from the EOLTAA scheme. We let *A*_1_ possesses the (*upk*, *usk*, *Cert*) of the EOLTAA scheme so that it can simulate the Vote oracle and Tally oracle for *A*_2_. Note that, even though *A*_1_ possesses (*upk*, *usk*, *Cert*) of the EOLTAA scheme it does not help *A*_1_ in breaking the IND-CCA security. *A*_1_ passes *Params* to *A*_2_ and completed the Register phase.

In the Training phase, *A*_2_ issues *v* as a vote query to *A*_1_ which is the Vote oracle from *A*_2_’s view. *A*_1_ sets *m* = *v* and encrypts *m* to produce *C*. Then, *A*_1_ generates *π* on *C* and returns *Bal* = {*C*, *π*} = *α* to *A*_2_. *A*_2_ issues *Bal* as a tally query to *A*_1_. *A*_1_ sets *α* = *Bal* and uses its Decrypt oracle to simulate Tally oracle for *A*_2_, that is, *A*_1_ issues *α* to Decrypt oracle to verify if *α* is valid. The Decrypt oracle returns the decryption result to *A*_1_, *A*_1_ extracts the validity result from the decryption result and returns the validity result either valid or invalid to *A*_2_.

At some point, *A*_2_ decides that the Training phase is over and starts the Identifying phase. *A*_2_ chooses *v*_0_ and *v*_1_ as the challenge and passes *v*_0_ and *v*_1_ to *A*_1_. *A*_1_ sets *m*_0_ = *v*_0_, *m*_1_ = *v*_1_ and selects a random bit *b* = {0, 1}. *A*_1_ computes *C*_*b*_ from *v*_*b*_ and generates *π*_*b*_ for *C*_*b*_. *A*_1_ sets *Bal*_*b*_ = *α*_*b*_ = {*C*_*b*_, *π*_*b*_} and delivers *Bal*_*b*_ as the challenge to *A*_2_. With a probability $\varepsilon^{\prime }\leq \frac{1}{2}+n\left(k\right)$, *A*_2_ outputs a correct guess *b*′ in return. *A*_1_ uses *A*_2_’s answer as its guess. Since *b*′ = *b*, *A*_1_ thus breaks IND-CCA security.

As *A*_1_ simulates the environment perfectly, we have *ε* = *ε*′ and *t* = *t*′ as required where *A*_1_ runs in time *t* while *A*_2_ runs in time *t*′.

#### 6.4.2 Anonymity

**Theorem 5**. *Let AUTHPKE* = {*Register, Authentication, Verification*} *be the secure event-oriented linkable and traceable anonymous authentication scheme and public key encryption scheme and let e-voting* = {*Register, Vote, Tally*} *be the secure e-voting scheme. If the underlying AUTHPKE is* (*t*, *q*_*a*_, *ε*)-*anonymous, then the e-voting scheme is* (*t*′, *q*_*v*_, *ε*′)-*secure against indistinguishability under chosen voter’s vote attack (IND-CVA), where*

$$\begin{array}{c} t=t^{\prime },q_{a}=q_{v},\varepsilon =\varepsilon^{\prime }\leq \frac{1}{2}+n\left(k\right) \end{array}$$ (5)

*q*_*v*_ *is the vote query, q*_*a*_ *is the authentication query*, *ε is the non-negligible advantage to break the anonymity in AUTHPKE, ε*′ *is the non-negligible advantage to break the IND-CVA in e-voting, n is a negligible function parameterised by the security parameter k*, *and t is the time required to complete the attack*.

*Proof.* Suppose that *A*_2_ is an Adversary who (*t*′, *q*_*v*_, *ε*′)-breaks the IND-CVA of e-voting scheme and *A*_1_ = *A*_*AUTH*_ where *A*_*AUTH*_ is the Adversary who (*t*, *q*_*a*_, *ε*)-breaks the anonymity of the AUTHPKE scheme. We show that AUTHPKE scheme is not (*t*, *q*_*a*_, *ε*)- secure. Hence, we show how *A*_1_ can use *A*_2_ to (*t*, *q*_*a*_, *ε*)-break the anonymity of AUTHPKE scheme. *A*_1_ runs *A*_2_ as a subroutine and simulates its attack environment.

The AUTHPKE Challenger passes *Params* and public, private key (*pk*_*e*_, *sk*_*e*_) to *A*_1_ where the (*pk*_*e*_, *sk*_*e*_) are from the PKE scheme. We let *A*_1_ possesses the public key and private key (*pk*_*e*_, *sk*_*e*_) of the PKE scheme so that it can simulate the Vote oracle and Tally oracle for *A*_2_. Note that, even though *A*_1_ possesses (*pk*_*e*_, *sk*_*e*_) of the PKE scheme it does not help *A*_1_ in breaking the anonymity security. *A*_1_ passes *Params* to *A*_2_ and completed the Register phase.

In the Training phase, *A*_2_ issues *v* as a vote query to *A*_1_ which is the Vote oracle from *A*_2_’s view. *A*_1_ sets *m* = *v* and encrypts *m* to produce *C*. Then, *A*_1_ generates *π* on *C* and returns *Bal* = {*C*, *π*} = *α* to *A*_2_. *A*_2_ issues *Bal* as a tally query to *A*_1_. *A*_1_ sets *α* = *Bal* and uses its Decrypt oracle to simulate Tally oracle for *A*_2_, that is, *A*_1_ issues *α* to Decrypt oracle to verify if *α* is valid. The Decrypt oracle returns decryption result to *A*_1_, *A*_1_ extracts the validity result from the decryption result and returns the validity result either valid or invalid to *A*_2_.

At some point, *A*_2_ decides that the Training phase is over and starts the Identifying phase. *A*_2_ passes v* as the challenge to *A*_1_. *A*_1_ sets m* = v*, encrypts m* to obtain *C*_*b*_ where *b* ∈ {0, 1}. *A*_1_ generates *π*_*b*_ on *C*_*b*_. *A*_1_ sets {*C*_*b*_, *π*_*b*_} = *α*_*b*_ = *Bal*_*b*_. *A*_1_ returns *Bal*_*b*_ as the challenge to *A*_2_. With a probability $\varepsilon^{\prime }\leq \frac{1}{2}+n\left(k\right)$, *A*_2_ outputs a correct guess *Bal*_*b*_ in return. *A*_1_ uses *A*_2_’s answer as its guess. Since *Bal*_*b*_ is valid, then *α*_*b*_ is valid, thus *A*_1_ breaks anonymity security.

As *A*_1_ simulates the environment perfectly, we have *ε* = *ε*′ and *t* = *t*′ as required where *A*_1_ runs in time *t* while *A*_2_ runs in time *t*′.

#### 6.4.3 Unforgeability

**Theorem 6**. *Let AUTHPKE = {Register, Authentication, Verification} be the secure event-oriented linkable and traceable anonymous authentication scheme and public key encryption scheme and let e-voting* = {*Register, Vote, Tally*} *be the secure e-voting scheme. If the underlying AUTHPKE is* (*t*, *q*_*a*_, *ε*)-*unforgeable, then the e-voting scheme is* (*t*′, *q*_*v*_, *ε*′)-*secure against existential unforgeable under chosen vote attack (EUF-CVA), where*

$$\begin{array}{c} t=t^{\prime },q_{a}=q_{v},\varepsilon =\varepsilon^{\prime }\leq \frac{1}{2}+n\left(k\right) \end{array}$$ (6)

*q*_*v*_ *is the vote query, q*_*a*_ *is the authentication query, ε is the non-negligible advantage to break the unforgeability in AUTHPKE, ε*′ *is the non-negligible advantage to break the EUF-CVA in e-voting, n is a negligible function parameterised by the security parameter k, and t is the time required to complete the attack*.

*Proof.* Suppose that *A*_2_ is an Adversary who (*t*′, *q*_*v*_, *ε*′)-breaks the EUF-CVA of e-voting scheme and *A*_1_ = *A*_*AUTH*_ where *A*_*AUTH*_ is the Adversary who (*t*, *q*_*a*_, *ε*)-breaks the unforgeability of AUTHPKE scheme. We show that AUTHPKE scheme is not (*t*, *q*_*a*_, *ε*)- secure. Hence, we show how *A*_1_ can use *A*_2_ to (*t*, *q*_*a*_, *ε*)-break the unforgeability of AUTHPKE scheme. *A*_1_ runs *A*_2_ as a subroutine and simulates its attack environment.

The AUTHPKE Challenger passes *Params* and public, private key (*pk*_*e*_, *sk*_*e*_) to *A*_1_ where the (*pk*_*e*_, *sk*_*e*_) are from the PKE scheme. We let *A*_1_ possesses the public key and private key (*pk*_*e*_, *sk*_*e*_) of the PKE scheme so that it can simulate the Vote oracle and Tally oracle for *A*_2_. Note that, even though *A*_1_ possesses (*pk*_*e*_, *sk*_*e*_) of the PKE scheme it does not help *A*_1_ in breaking the unforgeability security. *A*_1_ passes *Params* to *A*_2_ and completed the Register phase.

In the Training phase, *A*_2_ issues *v* as a vote query to *A*_1_ which is the Vote oracle from *A*_2_’s view. *A*_1_ sets *m* = *v* and encrypts *m* to produce *C*. Then, *A*_1_ generates *π* on *C* and returns *Bal* = {*C*, *π*} = *α* to *A*_2_. *A*_2_ issues *Bal* as a tally query to *A*_1_. *A*_1_ sets *α* = *Bal* and uses its Decrypt oracle to simulate Tally oracle for *A*_2_, that is, *A*_1_ issues *α* to Decrypt oracle to verify if *α* is valid. The Decrypt oracle returns the decryption result to *A*_1_, *A*_1_ extracts the validity result from the decryption result and returns the validity result either valid or invalid to *A*_2_.

At some point, *A*_2_ decides that the Training phase is over and starts the Forging phase. With a probability $\varepsilon^{\prime }\leq n\left(k\right)$, *A*_2_ outputs a guess Bal* to *A*_1_. *A*_1_ uses *A*_2_’s answer as its guess. Since Bal* is valid, then α* is valid, *A*_1_ thus breaks unforgeability security.

As *A*_1_ simulates the environment perfectly, we have *ε* = *ε*′ and *t* = *t*′ as required where *A*_1_ runs in time *t* while *A*_2_ runs in time *t*′.

### 6.5 Security of Li et al’s transformed e-cheque scheme

We have shown that the e-voting scheme proposed by Li et al. [16] possesses confidentiality, anonymity, and unforgeability as proven in Theorem 4, Theorem 5, and Theorem 6 respectively. Therefore, it is obvious that following from Theorem 1, Theorem 2, and Theorem 3 respectively, the transformed e-cheque scheme also enjoys the corresponding security properties and fulfills the security requirements of an e-cheque scheme.

## 7 Discussion

Our e-voting to e-cheque transformation also benefits from Li et al.’s generic construction. Specifically, one can replace their authentication scheme Φ with other candidates yet our transformation would work as expected. However, we note that except anonymity, Φ should also satisfy linkability and traceability [16]. Therefore, anonymous authentication schemes such as the password-authenticated key exchange protocols based on oblivious pseudorandom function [22] and multi-factor authentication protocol based on “Honeywords” and “Fuzzy-Verifier” [23, 24] are not readily applicable.

It is also interesting to explore the reverse transformation, that is, from an e-cheque scheme to an e-voting scheme. From our transformation, we know that the tallier and voter in an e-voting scheme is the bank and payer, respectively, in the resulting e-cheque scheme. While a voter needs to be anonymous to the tallier, a payer needs not be anonymous to the bank. In fact, Yeow et al. exploited this weaker anonymity requirement to instantiate an efficient e-cheque scheme from an e-auction scheme that does not protect the winning bidder’s anonymity [15]. Thus, we conjecture that to realise the reverse transformation, the underlying e-cheque scheme needs to possess an anonymity property that is stronger than IND-CIA. With that said, if there exists a generic approach to upgrade the IND-CIA security in an e-cheque scheme, e-voting schemes is equivalent to e-cheque schemes. We leave this as an open problem.

## 8 Conclusion

We presented a generic transformation from e-voting to e-cheque and showed that the transformed e-cheque scheme possesses the security properties of indistinguishability under chosen cheque attack (IND-CCEA), indistinguishability under chosen cheque’s information attack (IND-CIA) and existential unforgeability under chosen cheque’s information attack (EUF-CIA) if the underlying e-voting scheme is indistinguishability under chosen ballot attack (IND-CBAA), indistinguishability under chosen voter’s vote attack (IND-CVA) and existential unforgeability under chosen vote attack (EUF-CVA) respectively. Finally, we demonstrated the newly proposed transformation by deriving a concrete e-cheque scheme from Li et al.’s e-voting scheme as an instance.

## Supporting information

S1 Data (BST)

S2 Data (BIB)
